# Visceral obesity and anastomotic leakage rates in colorectal cancer: a systematic review and meta-analysis

**DOI:** 10.3389/fonc.2023.1224196

**Published:** 2023-08-21

**Authors:** Linchong Yu, Wenjiang Wu, Shijun Xia, Yue Li, Zhigang Xu

**Affiliations:** Shenzhen Hospital of Guangzhou University of Chinese Medicine, Shenzhen, China

**Keywords:** anastomotic leakage, visceral obesity, colorectal cancer, meta-analysis, surgery

## Abstract

**Background:**

Numberous studies have heatedly discussed whether obesity is a risk factor for anastomotic leakage (AL) because of the increasing number of colorectal cancer (CRC) cases and high incidence of CRC in patients with obesity.

**Objective:**

We aimed to explore the relationship between visceral obesity(VO) and AL after CRC surgery. The databases of Pubmed, Embase, and the Cochrane Library were searched for relevant data and articles published until November 1, 2022. We identified the difference in the incidence of AL after CRC surgery between patients with and without VO. The quality of included studies was evaluated using the Newcastle- Ottawa Scale, and odds ratio (OR) and 95% CI were used to assess the association between VO and AL.

**Results:**

This meta-analysis included 7 studies with 2,136 patients. The OR of patients with VO versus those without VO was 2.15 (95%CIs = 1.46–3.15, test for heterogeneity: P = 0.29, I^2 =^ 18%) based on the fixed-effect model in seven studies. Notably, the difference between the two groups was statistically significant (Z = 3.91 P < 0.0001). Patients with VO in the colon cancer group exhibited a higher incidence of AL (OR = 2.88, 95% CIs = 1.38–5.99, test for heterogeneity: P = 0.27, I^2 =^ 20%) than those in the rectal cancer group (OR = 2.74, 95% CIs = 1.13–6.65, test for heterogeneity: P = 0.20, I^2 =^ 38%). In the studies in the relevant literature, heterogeneity was low. Regarding patients with VO, four Asian studies reported increased morbidity due to AL (OR = 2.79, 95% CIs = 1.35–5.78, test for heterogeneity: P = 0.35, I^2 =^ 9%) compared with three non-Asian studies.

**Conclusions:**

Our findings confirmed the significant relationship between VO and AL. Thus, VO could be considered a reliable risk factor of surgery for colon cancer.

## Introduction

Anastomotic leakage(AL) is one of the most common postoperative complications of colorectal cancer(CRC) surgery that has concerned surgeons or patients for several decades. The incidence rate of AL in patients with CRC resection has been reported to be 1.2%–14.9% (1–7).

Eliminating the risk factor for AL is globally recognized as one of the effective ways to decrease the incidence of AL. In several studies, many risk factors, such as sex, neoadjuvant chemotherapy, anastomosis level from the anal margin, and operating time, have been linked to AL. In addition, numerous studies have heatedly discussed whether obesity is a risk factor for an AL because of the increasing number of CRC cases and high incidence of CRC in patients with obesity. Therefore, whether obesity has an impact on AL morbidity following CRC surgery remains debatable.

There are currently two measures to evacuate the extent of obesity in patients: BMI and VFA. Obesity has been linked to postoperative complication technical challenges after CRC surgeries. Moreover, it is often defined by a body mass index (BMI) of >25 (8) or 30 (9)kg/m^2^ in many studies. However, several studies have reported that BMI is not necessarily associated with visceral obesity (VO) (8, 10, 11) and does not always adequately reflect the regional fat distribution. Therefore, it is still debatable whether BMI is an effective tools for the preoperative assessment of the CRC procedure. According to various recent studies, VO is debased on abdominal CT scan at the level of L3–L4, and this finding is thought to be a better option for the prediction of the AL morbidity in the management of CRC surgery (12, 13). However, the impact of VO on the incidence of AL after CRC resection still remains inconclusive.

Thus, given the insufficient statistical power of the existing studies, The primary aim of our study was to incorporate results of the relevant comparative studies to examine the link between VO and AL following CRC surgery given that the existing studies lack appropriate statistical power.

## Materials and methods

This systematic review and meta-analysis is based on the Preferred Reporting Items for Systematic Reviews and Meta-Analyses statements. Given that the present study is a meta-analysis, neither informed consent from the patients nor approval from the institutional review board was necessary.

### Study strategy

The Pubmed, Medline, and Embase databases were searched for studies evaluating the association between VO and the AL following CRC surgery, for example, studies evaluating the effect of VO on CRC resection and those evaluating the effect of association between VO and AL on CRC surgery, from inception until November 1, 2022.

We used the combination of terms as follows: either MeSH terms or terms in title/abstract related to (“Abdominal Visceral” or “Retroperitoneal Fats” or “VO”or “obesity”) and (“anastomotic leak” or “anasotomosis leak” or “anastomosis leakage” or “AL”). Only studies published in English were considered. Additionally, we manually searched and obtained reference lists of retrieved articles and review articles. The quality of the included studies was evaluated using Newcastle–Ottawa Scale (NOS) score.

### Inclusion and exclusion criteria

Studies that met the following eligibility criteria were included following the Cochrane recommendations (PICOS schema): a) those discussing the association between the VO and the AL after CRC surgery (either open or laparoscopic); b) those discussing both VO group and non-VO groups; c) retrospective, prospective, or cohort studies included patients with CRC; d) those with clear definition of VO; e) those with clear data on both VO group and non-VO groups as well as AL.f) The study reported odds ration (OR) with a 95%CI: alternatively, the data were used to calculate OR. g) the primary outcome of this meta-analysis was to compared the morbidity of AL between VO groups and non-VO groups.

Exclusion criteria were as follows: studies involving non-human populations, review articles, experimental studies, case reports, and studies without controls were excluded. Moreover, studies on gastric and small intestinal surgery as well as emergency surgery were excluded.

### Data extraction

Two reviewers (L.Y. and SJ.X.) independently extracted the following parameters from each study: (i) journal title, author name and year of publication; (ii) population characteristics of studies; (iii) study design and inclusion/exclusion criteria; (iv) type of the surgery and the disease; (v) numbers of individuals in the obese groups and non-obese groups; and (vi) outcomes of AL.

Any disagreements were resolved by consensus between the two reviewers.

### Quality assessment

The Newcastle–Ottawa Scale (NOS) (14) was used to rate the quality of the included studies. The scale takes into account the selection of the study groups, comparability of the groups and determination of the exposure or the outcome of interest for both case–control and cohort studies. Two investigators (L.Y. and SJ.X.) conducted this evaluation. The high quality of the study was indicated by a total score of ≥6. **Table 1** shows the NOS for the seven studies.

**Table 1:** Characteristics of included studies.

Author, Country and publish year	Study design	Study Size and Anastomotic Leakage(Total/AL)	Definition of obesity	Disease	Type of surgery	AL in VO group and non VO group	NOS
Alessandro Giani M.D; Italy; 2020 (15)	Prospective cohort study	122/10	Unmentioned	Rectal Cancer	Open and Laparoscopic	6 AL:30 VO 4AL:92 nonVO	8
Hamit Cakir, MD; The Netherlands; 2015 (16)	Retrospective cohort study	564/36	VFA of >100 cm^2^	Colon Cancer	Open and Laparoscopic	29 AL:367 VO 7AL:197 nonVO	8
Wei-Zhe Chen; China 2018 (17)	Prospective study	376/16	VFA>130 cm^2^ (Men) VFA>90 cm^2^ (Women)	Colorectal Cancer	Open and Laparoscopic	12 AL:191 VO 4 AL:185 non VO	8
Jeonghyun Kang; Korea; 2011 (18)	Prospective study	142/12	VFA≧130 cm²	Rectal Cancer	Laparoscopic	4 AL:29 VO 8 AL:113 nonVO	7
Ishii; Japan; 2005 (19)	Prospective study	46/6	VFA>100 cm^2^	Rectal Cancer	Laparoscopic	0 AL:9 VO 6 AL:37 nonVO	6
Jun Watanabe; Japan; 2013 (20)	Prospective study	338/11	VFA≧100 cm^2^	Colon Cancer	Laparoscopic	9 AL:144 VO 2 AL:194 nonVO	7
Paulina M.V; Mexico; 2021 (21)	Retrospective study	548/53	VFA>100 cm^2^	Colorectal Cancer	Open and Laparoscopic	33 AL:295 VO 20 AL:253 nonVO	7

### Outcomes

The primary aim of our analysis was to combine the result of comparative studies to determine the relationship between VO and AL after CRC surgery.

### Statistical analysis

All statistical analyses were performed using the Review Manager (RevMan) version 5.4 provided by Cochrane Collaboration. The values of ORs with 95% CIs were pooled using generic inverse variance methods for describing dichotomous variables. Heterogeneity was evaluated using the statistical values of Q and I^2^. For P < 0.1 or I^2^ > 50%, heterogeneity was considered to be present, and the random-effects model was performed. Alternatively, the fixed-effects model was performed. P–values of < 0.05 were considered statistically significant.

## Result

**Figure 1:**
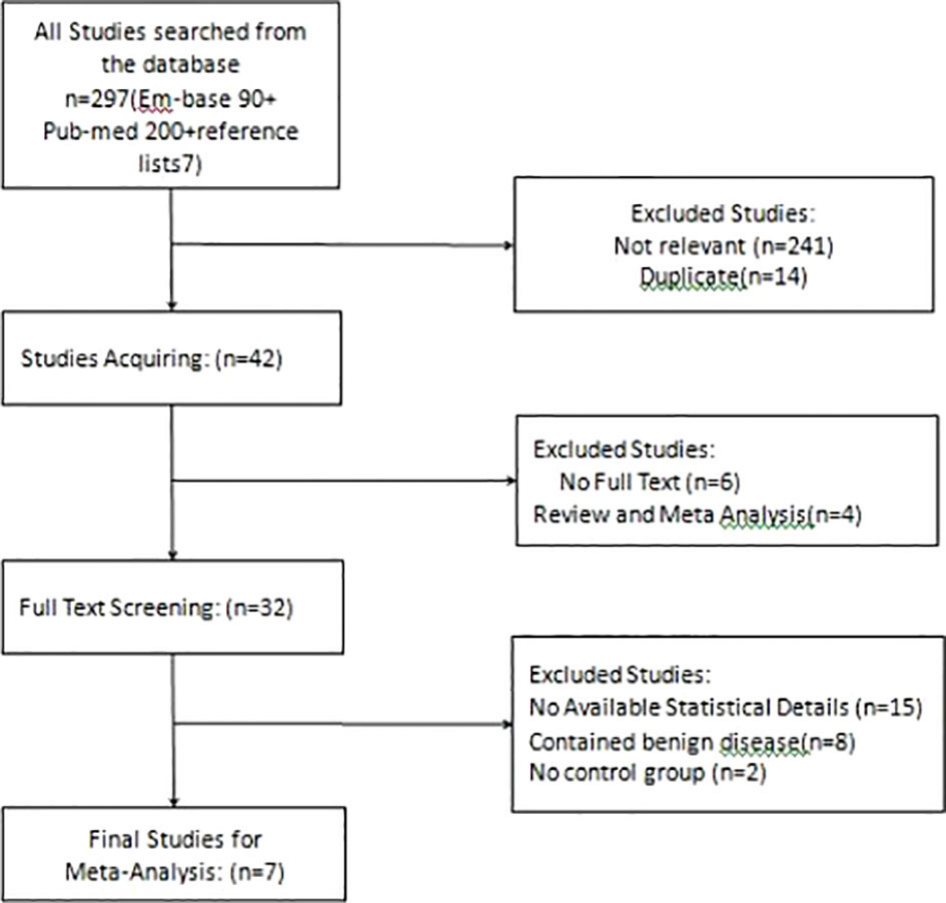
Flowchart of the selection process.

The selection procedure has been shown in the **Figure 1**. The database search yielded 297 studies. Of these, seven studies were ultimately included after exclusion based on the inclusion and exclusion criteria (15–21).

The characteristics of the included studies are summarized in **Table 1**. We identified seven studies published during 2006-2021 involving 2136 patients with 144 leakages. Among these studies, 1035 patients with visceral obesity (visceral fat area [VFA] of ≥100 cm^2^ or 130 cm^2^) and 1101 patients with nonvisceral obesity were included. Of those with visceral obesity, 87 (8.41%) and 948 (91.59%) cases were associated with and without AL.

**Table 1** presents information about the seven studies, including that about the year of publication, country, types of the study design, definition of VO, type of surgery and disease, and specified controls for patient with obesity. Notably, three papers were of retrospective studies, whereas the others were of prospective studies. Of the seven studies, four published from 2005 to 2018 were from Asia, one was from North America, and two published from 2015-2020 were from European institutions. Although the remaining studies reported clear data to calculate the ORs with 95% CI, only three (16, 20, 21) of the included studies directly published the ORs with 95% CI.

Two measures that are most commonly used to evaluate an individual’s level of obesity are BMI and VFA. A single-slice CT scan was used to quantify VFA in the current meta-analysis at the level of the navel (L3–L4) (22, 23). Additionally, many studies used alternative cutoffs, such as 130 cm^2^ (24), 100 cm^2^ (23), and 90 cm^2^ (25), to define VO and treat VFA as a dichotomous variable.

Details of the numerous definitions of VO are summarized in **Table 2**. The study from Mexico (21) only used the VFA rather than BMI to describe the obesity as its objective was to evaluate the relationship between skeletal muscle index and VFA with 30-day mortality in CRC surgery. Four Asian studies used VFA to describe the VO, whereas the specific cutoffs for diagnosing VO (VFA > 100 cm^2^) were different between two Japanese studies and the Chinese and Korean study according to the VFA cutoff line of the Japan Society for the Study of Obesity. Although most studies simply use same cutoff for both males and females, Chinese studies use distinct cutoffs for males and females. Moreover, the number of the lumbar CT scan is different among seven studies. Korean studies defined obesity as VFA of >130 cm^2^, whereas other five studies defined it as VFA of > 100 cm^2^.

**Table 2:** Details of the various definitions of VO.

Visceral Obesity			Reference
Parameter	Tool	Cut off	
VFA	Not Mention	Not Mention	Alessandro Giani M.D; Italy; 2020
VFA	CT:L3-L4 -140to-50 Hu	>100cm^2^	Hamit Cakir, MD; Netherlands; 2015
VFA	CT:L3 -150to-30 Hu	M>130cm^2^ F>90cm^2^	Wei Zhe Chen; China 2018
VFA	CT:L4-L5	>130cm^2^	Jeonghyun Kang; Korea; 2011
VFA	CT:L3-L4	>100cm^2^	Y.Ishii; Japan; 2005
VFA	CT:L4 -190to-30 Hu	≥100cm^2^	Jun Watanabe; Japan; 2013
VFA	CT:L3 -190to-30 Hu	>100cm^2^	Paulina M.V; Mexico; 2021

**Figure 2:**
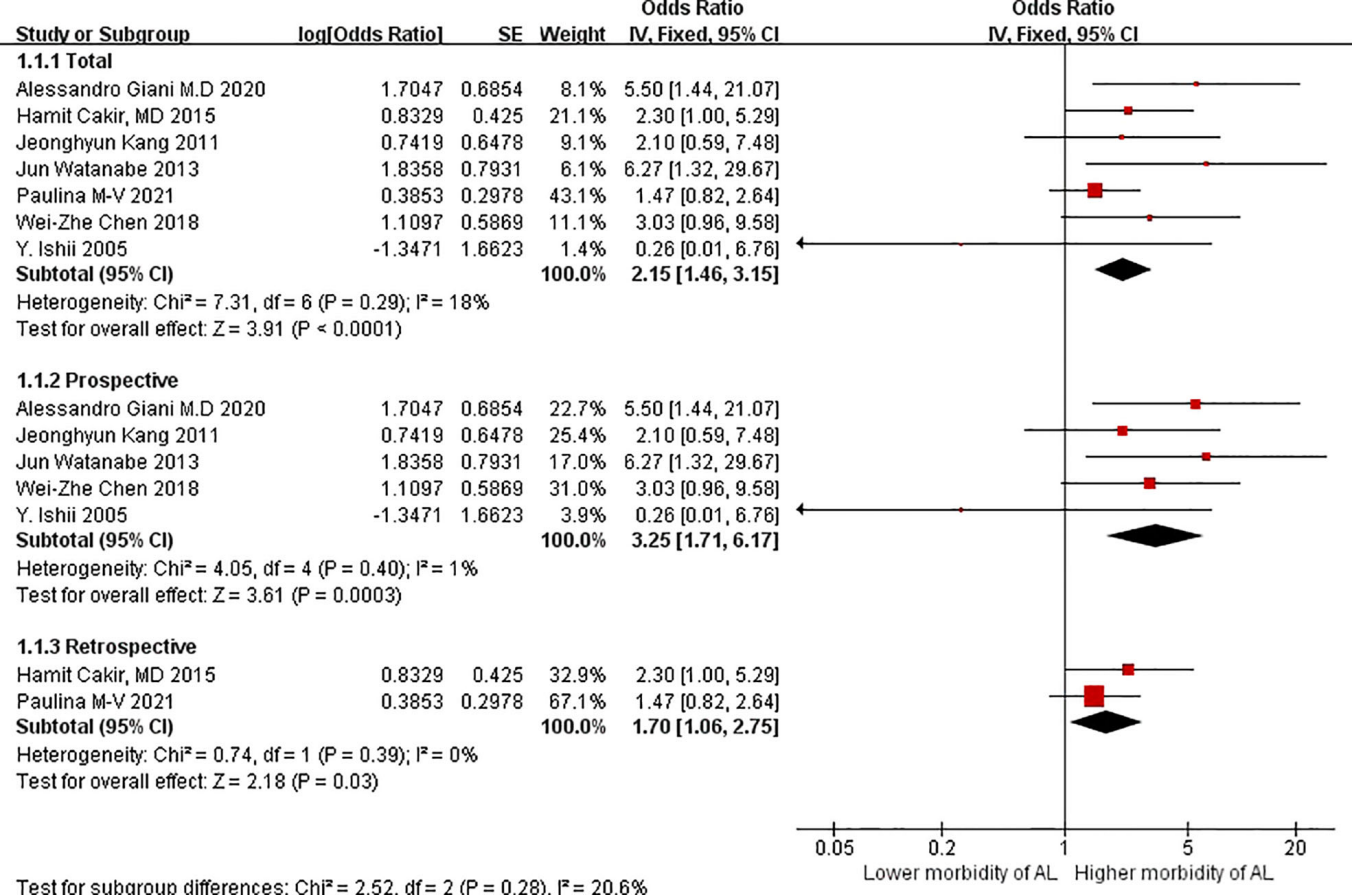
Shows a forest plot comparing the incidence of AL in visceral and non-visceral groups.

The OR of patients with VO versus without VO was 2.15 (95%CIs = 1.46–3.15, test for heterogeneity: P = 0.29, I^2 =^ 18%; **Figure 2**) based on the fixed-effect model in seven studies; the difference between the two groups was statistically significant (Z = 3.91 P < 0.0001).

Regarding subgroup analyses in terms of study design, the OR of the patients with VO versus those without VO was 3.25 (95%CIs = 1.71–6.17, test for heterogeneity: P = 0.40, I^2 =^ 1%; **Figure 2**) based on the fixed-effect model in prospective studies. The OR in terms of VO in the subgroup analyses was 1.70 (95%CIs = 1.06–2.75; **Figure 2**) based on the fixed-effects model in retrospective studies, without significant heterogeneity (P = 0.39, I^2 =^ 0%).Similarly, difference between the two groups were statistically significant in terms of prospective studies and retrospective studies.

**Figure 3:**
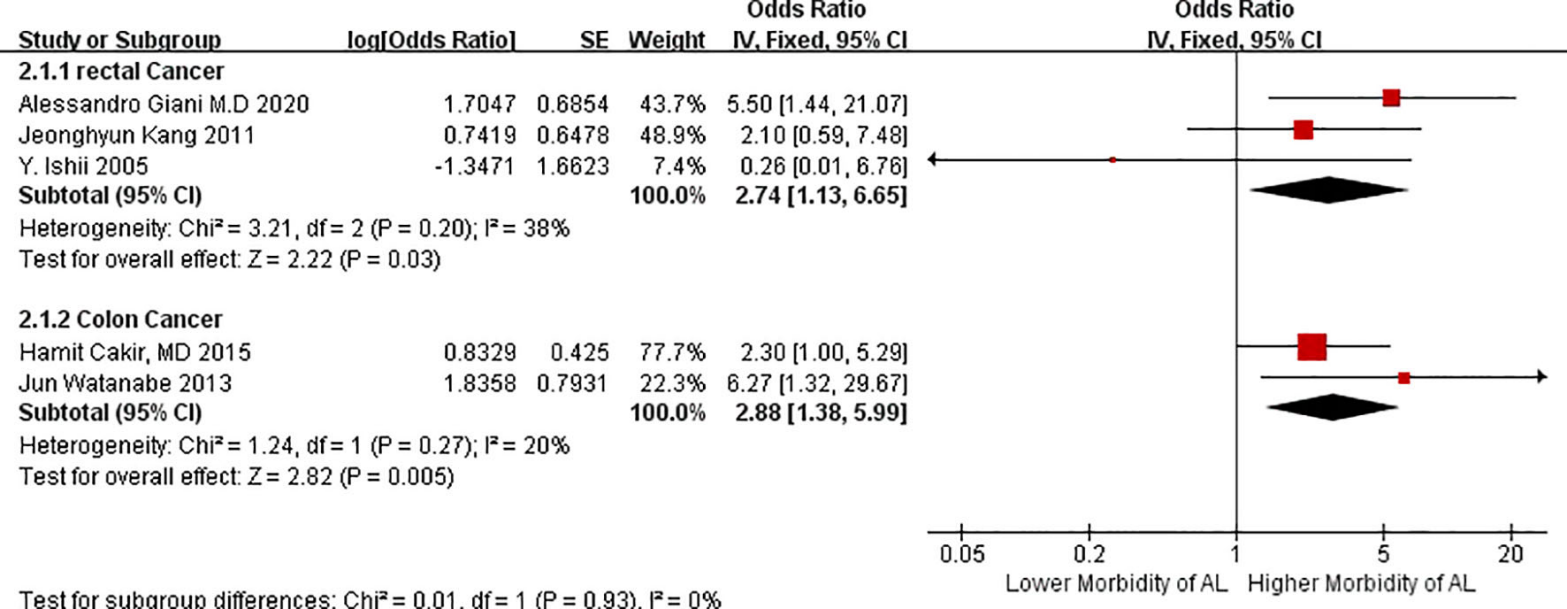
Forest plot of AL morbidity in patients with visceral obesity stratified by the factor of different location of cancer.

We divided AL risk in patients with VO according to the factor of colon and rectal cancers because these cancers may have different effects on the outcomes of AL. Patients with VO have a higher incidence of AL in the colon cancer group (OR = 2.88, 95%CIs = 1.38–5.99, test for heterogeneity: P = 0.27, I^2 =^ 20%, **Figure 3**) than those in the rectal group (OR = 2.74, 95%CIs = 1.13–6.65, test for heterogeneity: P = 0.20, I^2 =^ 38%, **Figure 3**). Heterogeneity between the two groups was low. This suggest that VO is be a reliable tool for predicting morbidity due to AL in cases of major intestinal CRC, particularly cancer localized in the colon.

**Figure 4:**
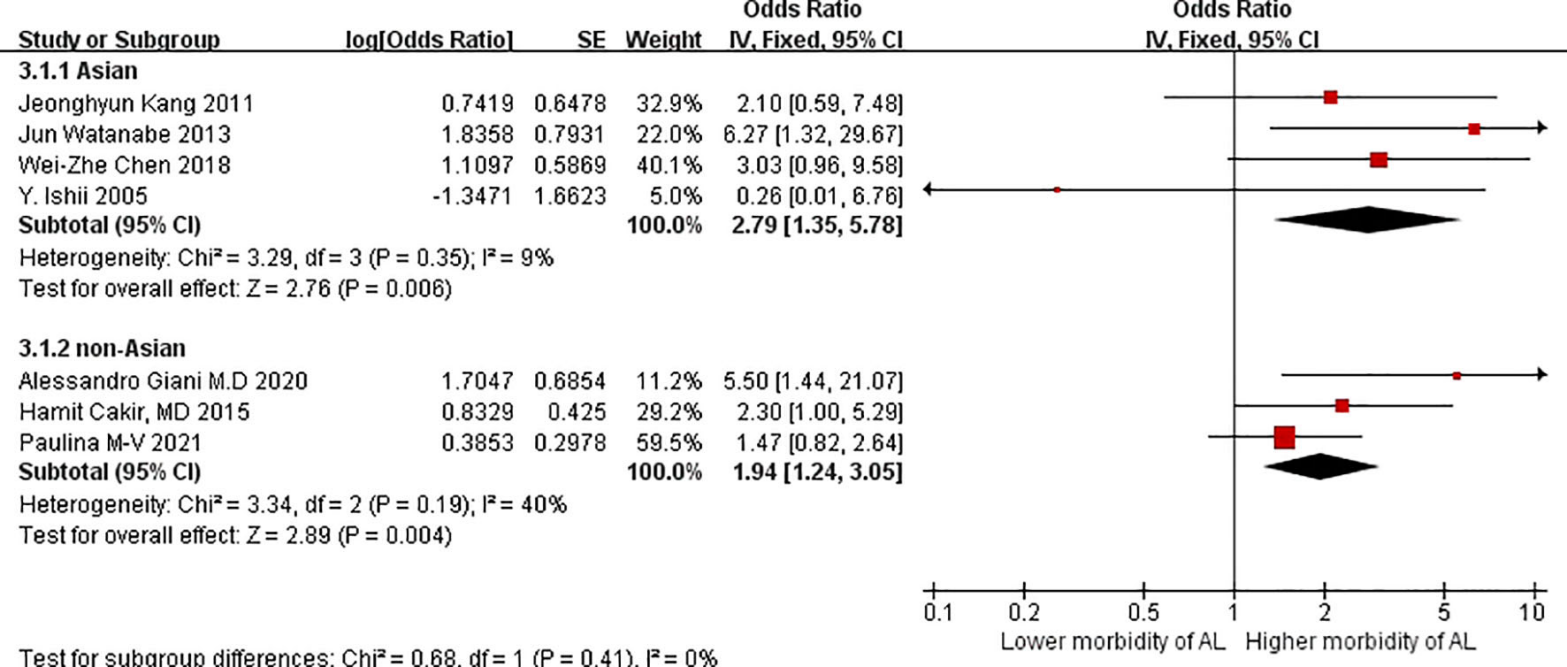
Forest plot of AL morbidity in patients with VO stratified by Asian and non-Asian studies.

Regarding patients with VO, four Asian studies reported higher morbidity due to AL (OR = 2.79, 95%CIs = 1.35–5.78: test for heterogeneity: P = 0.35, I^2 =^ 9%; **Figure 4**) compared with three non-Asian studies. Although Moon HG et al. (26), Cecchini S et al. (27), and Ballian N et al. (28) reported no increase in morbidity among patients with VO, nevertheless, we believed that VO is better suitable for Asian patients regarded as an assessment for morbidity due to AL.

### Publication bias and sensitive analysis

In accordance with the Cochrane Handbook for Systematic Reviews of Interventions, we did not examine publication bias because the number of the included studies was <10. Sensitivity analysis was performed to evaluate the stability of the results in subgroup of Rectal cancer and Non-asian, resulting in the omission of one study from the meta-analysis at a time. The results revealed no significant change in the group of Non-asian. However, we discover the heterogeneity decreased after the removal of Y. Ishii in the group of Rectal cancer, because the number of total cases and AL cases in Y. Ishii were lower than those of the other two articles, and there was no case of AL in the VO group in Y. Ishii. Thus, the result of Y. Ishii had a great impact on the heterogeneity in subgroup of Rectal cancer. With Y. Ishii excluded, we derive the result showing a large correlation between VO and AL in the subgroup of Rectal cancer. The results of sensitivity analysis are presented in **Table 3**.

**Table 3:** Sensitivity analysis results after removing one study at a time.

A univariate analysis for the association between VO and AL in rectal cancer				
Removed study	OR	95% CI	P_heterogeneity_	I^2^
Alessandro Giani (2020) (15)	1.59	0.49-5.20	0.44	27%
Jeonghyun Kang (2011)	3.53	1.02-12.22	0.05	65%
Y.Ishii (2005) (19)	3.31	1.31-8.32	0.01	4%
None	2.74	1.13-6.65	0.03	38%
B univariate analysis for the association between VO and AL in non-asian subgroup				
Removed study	OR	95% CI	P_heterogeneity_	I^2^
Alessandro Giani 2020 (15)	1.70	1.06-2.75	0.03	0%
Hamit Cakir 2015 (16)	1.81	1.06-3.10	0.03	68%
Paulina M-V 2021 (21)	2.93	1.44-5.95	0.003	14%
None	1.94	1.24-3.05	0.004	40%

## Discussion

Obesity is a global health issue that impacts the surgical outcome of patients with CRC as a risk factor (29–35). Both BMI and VFA could be considered as measures of obesity in humans. Specifically, VO was defined as an intra-abdominal VFA of >100 cm^2^ in a horizontal slide in the umbilical plane on a CT-scan at the level of the navel (L3–L4) whereas general obesity was defined as BMI of >30kg/m^2^ based on height and weight. Moreover, the cutoff line of the VFA varies for Asian and European populations. Based on the review of the seven papers in the present study, studies in China (17) use two separate cutoff lines for male and female patients, but other studies in other countries only use one cutoff for patients with VO. Two distinct cutoff lines (130 cm^2^ and 100 cm^2^), four different planimetric cross-sectional scan levels of CT (L3–L4, L3, L4, L4–L5) and four different attenuation ranges (–190 to –30, –140 to –50, –150 to –50, –150 to –30 HU) were utilized in all studies, except for one. According to the result of our study, there is some agreement on the cutoff line for BMI obesity owing to differences in obesity prevalence and body fat distribution between Asian and European populations; however, there is still disagreement about the criteria of the cutoff line and the plane of the planimetric cross-sectional scan level for VFA in defining VO. We could not analyze the differences between the various of scale listed above in the current study because of the small number of total investigations. Accordingly, it is critical to establish an appropriate VFA cutoff line, cross-sectional scan level and attenuation range for all ethnics in the preoperative assessment and future studies.

Excessive peri-visceral adipose tissue, a disproportionately large omentum and a thickened mesentery are observed in patients with VO. In these cases, VO—as opposed to subcutaneous deposits—has been postulated as a superior predictor of technical operating difficulty and patient outcomes (12, 36). In case of rectal cancer surgery, the technical difficulties associated with surgery in the narrow pelvis may be better reflected by VFV than BMI. As an intraoperative factor, intra-abdominal fat increases the technical challenges leading to compaction of VO to AL rates. Obesity causes chronically increased intra-abdominal pressures (37), which could impair micro-circulation of the anastomosis. In addition, obesity is associated with metabolic abnormalities, which cause an inflammatory state that may have a negative effect on normal tissue repair and anastomotic healing (38, 39). However, some (12, 13, 16, 40) studies reported that although BMI (linearly correlated with BMI) cannot explain intra-abdominal adiposity, VO can.

We investigated the effect of VO on rates of anastomosis leakage following laparoscopic or open surgery for CRC in the current meta-analysis, which included seven studies that used the VFA value as a method to determine VO for different groups of patients. The curent meta-analysis sheds light on the controversially reported association between VO and AL risk in CRC surgery. Our analysis of the AL result revealed that VO was significantly associated with increased morbidity due to AL; this finding is consistent with other prior studies (16, 18, 41, 42).

Many studies found no significant link between obesity and the development of AL after the surgery for rectal cancer (2, 17, 43–45). However, some studies reported that the measuring visceral fat on a CT scan is a more sensitive factor than BMI in predicting the development of anastomotic dehiscence (16, 20, 32, 34, 35, 46). In contrast, some studies on CRC surgery identified a remarkable association between obesity and AL (16, 20, 42, 47,). According to our findings, the VO group exhibited a greater incidence of AL after both rectal and colon cancer surgery. Thus, it is convincing that VFA, which is strongly associated with a higher incidence of AL, is a reliable tool for analyzing the effects of adiopse tissue deposition on incidences of AL. Obesity may be a risk factor for leaks in extremely low rectal anastomosis because it may be related to tension at the anastomotic site, but technical challenges associated with surgery in the narrow pelvis may be better reflected by VFV(visceral fat volume) than BMI. These could be some of the reasons why VO is superior to BMI obesity in terms of the association with the morbidity of AL.

Our study suggests that VO is related to AL in CRC surgery, so preoperative evaluation of patients’ VO by CT may help the surgeon to acknowledge the morbidity of postoperative AL and develop relevant preoperative strategies to reduce the morbidity of AL. Since it’s unrealistic to change the situation of VO before the surgery in the short term, the only thing we can to is to prevent and intervene AL from other perspectives. For example, it is still common practice to place a drainage tube near the anastomosis or drainage in the colon and pelvic cavity, and reducing the time of using the cutting closure device is also considered to reduce the incidence of AL after laparoscopic rectal cancer (48). The fluorescent angiography (indocyanine green-fluorescence angiography, ICG-FA) is a detection technology that can be detected by the imaging system and can detect the insufficient blood supply of the anastomosis early. After ICG-FA, the transection line can be transferred to the site of good blood perfusion, and the anastomosis here can ensure the blood supply of the anastomosis (49). To reduce the incidence of CRC postoperative AL measures above, we strongly believe that prevention is more meaningful than treatment. As for low location rectal cancer surgery, preventive ileostomy or in anastomosis local use absorbable suture reinforcement can be regarded as effective measures, but the disadvantage of preventive stoma is possible to bring preventive stoma complications and the second operation. The early appearance of AL means the failure of the suture procedure, resulting in an immediate rupture of the anastomosis. The advanced AL is more likely caused by the physiological condition and the vulnerability of anastomotic tissue of the patients, which are the crucial point and bottleneck problem of reducing the incidence of AL from our point of view. There is no optimal treatment for AL and the associated high morbidity and fatality, but with further study and improvement of variable risk factors, the care of preoperative CRC patients will be best optimized (50).

To the best of our knowledge, this is the first study to compare the relationship between VO and morbidity due to AL in patients who had undergone CRC surgery for malignant tumors. The study has some limitations. First, some of the included studies were retrospective; thus, the final result could have been influenced by confounding factors. Nevertheless, all the studies included in this meta-analysis were rated as high quality. Second, as several studies included data on different cancer foci as well as different ethnicities, we tended to address this issue by performing a stratified analysis depending on these criteria. Third, the cutoff of the VFA can differ between studies depending on the research institution from different locations. Fourth, owing to the unclear group data, we could not examine the relationship between obesity and AL across different types of the surgery (open or laparoscopic). Fifth, studies analyzing the influence of VO on the outcome of AL in patients with CRC are insufficient; hence, only seven studies meet our eligibility standards. In future, risk analyses for AL in CRC surgery should include visceral fat levels and account for subgroup differences.

## Conclusion

In the current study, we investigated the relationship between VO and morbidity due to AL following surgery for CRC. Our findings confirmed the remarkable relationship between VO and AL. Furthermore, the incidence of AL after the surgery for colon cancer in patients with VO was high in all cancer groups, demonstrating that VO maybe a valid risk factor of surgery for colon cancer.

## Data availability statement

The original contributions presented in the study are included in the article/supplementary material. Further inquiries can be directed to the corresponding author.

## Author contributions

First author: LY (analysis and interpretation of data, drafting the article or revising it critically for important intellectual content). The corresponding author: WW (conception and design, final approval of the version to be published). Other author: SX, ZX, and YL (acquisition of data). All authors contributed to the article and approved the submitted version.
